# Olive Polyphenols and the Metabolic Syndrome

**DOI:** 10.3390/molecules22071082

**Published:** 2017-06-29

**Authors:** Bandhita Saibandith, Jeremy P. E. Spencer, Ian R. Rowland, Daniel M. Commane

**Affiliations:** Hugh Sinclair Unit of Human Nutrition, Department of Food and Nutritional Sciences, School of Chemistry Food and Pharmacy, University of Reading, Whiteknights, Reading RG6 6AH, UK; b.saibandith@pgr.reading.ac.uk (B.S.); j.p.e.spencer@reading.ac.uk (J.P.E.S.); i.rowland@reading.ac.uk (I.R.R.)

**Keywords:** central obesity, dyslipidaemia, hyperglycaemia, hypertension, metabolic syndrome, olive polyphenols

## Abstract

Here, the effects of consuming polyphenol-rich olive products, including olive leaves, their crude extract, and extra virgin olive oil, on aspects of the metabolic syndrome are reviewed. We have sought to summarize the available scientific evidence from dietary intervention trials demonstrating a role for these phytochemicals in ameliorating aberrant glucose metabolism, high blood pressure and elevated blood lipids, and we discuss the potential mechanisms underpinning these observations. Searches for relevant literature published in English were conducted via PubMed and Science Direct. Based on published dietary intervention studies, there is convincing evidence to show that olive polyphenols, independently of olive lipids, reduce risk factors for metabolic syndrome, in particular by improving blood sugar and blood pressure control, and in reducing low density lipoprotein oxidation. There is more limited evidence to suggest that the consumption of olive polyphenols or related products can reduce body weight and visceral fat or impede weight gain, and similarly there are some limited data suggesting improved lipid profiles. There is some mechanistic data to support observations made in human volunteers, but further work is needed in this area. The consumption of olive polyphenols within the context of a healthy pattern of food intake may, in part, explain the reduced risk of metabolic disease associated with adherence to the Mediterranean diet.

## 1. Introduction

The metabolic syndrome (MetS) is characterised by a cluster of interrelated markers of ill health, including obesity, hyperglycaemia, dyslipidaemia and hypertension [1]; up to 25 percent of the world’s adult population may satisfy the criteria for this syndrome [2]. Individuals with the MetS carry a five-fold greater risk of developing type 2 diabetes [3] and a two-fold increase in the risk of the developing subsequent cardiovascular disease [4]. It is generally accepted that the MetS is related to modifiable behavioural risk factors, including unhealthy patterns of food consumption, physical inactivity, smoking, and alcohol abuse [5,6].

Dietary guidelines consistently emphasise the importance of placing plant-based foods at the core of a healthy pattern of food consumption [7]. This predominance of plant foods is a defining feature of the Mediterranean dietary pattern, which is also characterised by a high consumption of olive products. Adherence to the Mediterranean diet is associated with a reduced risk of MetS [8]. There may be specific benefits arising from olive polyphenols within the context of the Mediterranean diet [9,10,11,12,13]. The most well studied bioactive small molecules present in olive products are the catecholic compounds, oleuropein (OL) and hydroxytyrosol (HT). Both OL and HT have established bioactivity, with demonstrable experimental antioxidant [14], anti-inflammatory [15], and antimicrobial properties [16]. These compounds are thus credited with some of the health benefits observed in those consuming high quantities of olive foods [17,18]. The current review will present the evidence from intervention studies illustrating the relationship between the chronic consumption of polyphenol-rich olive products and aspects of the MetS. It will also discuss the potential mechanisms linking olive polyphenol consumption to improvements in markers of health.

## 2. Phenolic Content of Olive Products

The olive tree is a valuable source of polyphenols [19]; these are present in the plant as secondary metabolites to combat pests, bacterial infection and other stresses [20]. Olive oil is a product of the mechanical extraction of the olive fruit; compositionally it is an oleic acid, mono unsaturated fatty acid (MUFA), rich product with a lesser (0.4–5%) non-triglyceride component [21]. Olive polyphenols are found in both the lipid and the water fractions of olive oil [22]. The total phenol content in olive oil varies between oils, according to crop and harvest conditions and according to processing, storage, and preservation methods [23,24]. In refined oils, phenolics may be present at concentrations of approximately 62 ± 12 mg/kg, whereas in high quality extra virgin olive oils (EVOO) this concentration may be much higher [10]. The olive leaf has even higher concentrations of total phenolic compounds relative to the olive fruit and olive oil; 1350 mg/kg fresh leaf [25] versus 232 ± 15 mg/kg of EVOO [22,26]. A number of olive leaf extracts (OLE) are available to consumers and are marketed as food supplements or nutraceuticals containing concentrated amounts of phenolics (range 1057–4831 mg/kg dry extract) [27]. The possibility of using phenolics extracted from olive leaves to enrich edible oils is also being explored [28,29]. OL is the most abundant phenolic compound present in olive leaf, seed, pulp and peel of unripe olives [19,30]. During fruit maturation, OL undergoes hydrolysis, yielding different products, including (2-(3,4-dihydroxyphenyl)ethanol) (HT), which is the major phenolic compound in EVOO (14.42 mg/kg) [25,31]. Other phenolics, namely rutin, caffeic acid, verbascoside, and the flavone-7-glucosides of luteolin and apigenin, are also present in olive products but in lower concentrations [9,32]. The radical scavenging activity of some of these minor phenolics may also be very high [21], and it is feasible that the combination of phenolics, when consumed in a food or a crude extract, has a greater influence on the health of the consumer than would a purified individual compound.

## 3. Effects of Olive Polyphenols on the Main Features of Metabolic Syndrome

The manuscripts evaluated for the purposes of this review were identified using the following key search words: olive phenolics, olive leaf phenolics, olive oil phenolics, HT, and OL. Abstracts were screened and papers were selected for this review where there was a dietary intervention involving the delivery of olive polyphenols and where the endpoints were related to the metabolic syndrome, i.e., obesity, hyperglycaemia, hypertension, lipid peroxidation, hyperlipidaemia cardiovascular disease, diabetes mellitus. A summary of the identified studies is provided in Table 1.

### 3.1. Central Obesity

A progressive increase in total adiposity is associated with insulin resistance and all the other components of the MetS, including increased blood pressure, glucose, and lipid concentrations [49]. The consumption of foods rich in polyphenols, including green tea catechins, resveratrol and curcumin, is weakly associated with anti-obesogenic effects [50]. Further, the Mediterranean dietary pattern (MedDiet) itself is associated with a lower risk of obesity despite the relatively high consumption of energy rich olive oils [51,52]. In a follow-up of a large Spanish cohort (The SUN project, Seguimiento Universidad de Navarra) high intakes of olive oil were not associated with increased risk of subsequent weight gain (Table 1) [53]. At least three long-term human dietary intervention studies with the Mediterranean diet plus a high intake of olive oil have demonstrated a reduction in body mass [42,43,47]. The Mediterranean diet has a comparatively lower energy density than the standard western diet; however, studies in animal models fed polyphenol-rich olive extracts suggest a further contributing role for olive phenolics in body weight management. In one study, mice were randomly divided into groups that received either a chow diet, a high-fat diet (HFD), or a 0.15% OLE-supplemented diet for eight weeks. The OLE-fed mice showed significantly reduced body weight gain, and had lower visceral fat-pad mass, compared to the HFD-fed mice [54]. Elsewhere mice fed a 0.03% OL-supplemented HFD for 10 weeks showed reduced body weight gain (−55%) compared to the control [55]. Furthermore, HT supplementation of 10 mg/kg/day prevented HFD-induced obesity in mice at 17-weeks [56]. The addition of rutin [57], luteolin [58], caffeic acid [59] and apigenin [60] to the diets of experimental animals have all been shown independently to ameliorate weight gain, liver, and adipose tissue mass. Unfortunately, human dietary intervention studies with polyphenol-rich OLE or with isolated olive phenolics have typically been relatively short in duration, and show no demonstrable reduction in body-weight reported.

Despite the paucity of human data showing a direct effect on either weight loss or the attenuation of weight gain, potential mechanisms through which olive polyphenols might influence body composition have been suggested. In experimental models olive phenolics inhibit lipid and carbohydrate digestion in the gut [61], thereby suppressing macronutrient absorption and uptake [37]. In addition, in vitro and in vivo models have suggested that olive polyphenols can inhibit pre-adipocyte differentiation, suppress lipogenesis, induce lipolysis, and promote adiponectin secretion [62,63,64], possibly mediated through the suppression of adipogenic gene expression (PPARγ, C/EBPα, CD36, FAS, and leptin) at the mRNA and protein levels [54,58,60,65,66,67].

### 3.2. Hyperglycaemia

Impaired carbohydrate metabolism and insulin resistance are defining features of the MetS [4]. Polyphenol-rich foods, including tea, cocoa, cinnamon, grapes, and berries, are reported to modulate carbohydrate metabolism, and attenuate hyperglycaemia and insulin resistance [68,69]. Dietary supplementation with olive polyphenols has been shown to exert an anti-hyperglycaemic response in animal models [61,70,71]. For example, the consumption of a phenolic-rich olive extract significantly decreased serum glucose in alloxan-induced diabetic rats after a four week intervention with a dose of extract equivalent to 8 mg/kg OL and 16 mg/kg HT [72]. Similarly, diabetic rats consuming 0.5 mg/kg OLE for 30 days showed improved blood glucose, and increased plasma insulin [73]. These observations are mirrored in a study in obese diabetic mice, which also showed improved glucose control when fed an OL-enriched diet independently of any change in body weight [74]. Further, in a rabbit model of diabetes, the consumption of extracted OL (at 20 mg/kg body weight) reduced blood glucose after 16 weeks compared to the controls [75].

More importantly these anti-hyperglycaemic effects are also observed in human dietary intervention studies in volunteers with pre-existing elevated blood sugar [13,32,37,76]. For example, de Bock et al. demonstrated that supplementation with olive leaf extract for 12 weeks (51.1 mg OL, 9.7 HT per day) was associated with a 15% improvement in insulin sensitivity in overweight middle-aged men (*n* = 46) [32]. An intervention study in recent-onset type-2 diabetic patients consuming 500 mg of OLE once daily led to improved long-term sugar control as evident by a reduction in glycated haemoglobin (HbA1c) after 14 weeks [37].

The minor phenolics may also contribute to the observed anti-hyperglyceamic response. Experimental supplementation with luteolin [77] or apigenin [78] has been shown to significantly decrease insulin resistance in diet-induced obese mice. Additionally, a dietary intervention with 500 mg daily of supplementary rutin reduced fasting glucose levels by over 10% in diabetic patients at 30 and 60 days compared to baseline; this was possibly coupled to a concurrent reduction in body weight [79].

Potential mechanisms underpinning the anti-hyperglycaemic activity of dietary polyphenols have been proposed, In vitro studies show the inhibition of amylase and α-glucosidase, in the gut this would result in the suppressed digestion of starch and therefore a lower glycaemic response to foods [80,81,82]. Other studies have suggested that polyphenols exert a direct suppression of the proteins involved in the intestinal transport of dietary carbohydrate; this has previously been reviewed [69]. Incidentally, green tea phenolics have been credited with directly stimulating insulin-mediated glucose uptake in myocytes which would improve plasma glucose control [83]; this has not, as yet, been demonstrated for the olive phenolics and certainly not for their circulatory plasma metabolites.

### 3.3. Hypertension

Adherence to the Mediterranean diet is inversely associated with both systolic and diastolic blood pressure [84]. Dietary interventions with extra virgin olive oil show marked improvements in blood pressure in hypertensive and pre-hypertensive volunteers. For example, in a crossover study in which medicated hypertensive volunteers in the south of Italy were asked to replace their fats with either EVOO or sunflower oil, The EVOO led to a reduction in blood pressure (BP), and for some, a decreased reliance on anti-hypertensive medication [40]. Similarly, in a dietary intervention in an overweight and obese U.S. cohort, BP was markedly lowered when the source of fat was replaced with EVOO [41]. Moreno-Luna et al. compared the effect of interventions with polyphenol-rich olive oil and high-oleic sunflower oil (polyphenol-free) in hypertensive women; the olive oil rich-diet significantly reduced both systolic blood pressure (SBP) and diastolic blood pressure (DBP) [48]. Further, Fito et al. reported a decrease in SBP after high-phenolic olive oil consumption but not with a low-phenolic refined olive oil, when consumed by hypertensive stable coronary heart disease patients [45]. These studies collectively suggest that the hypotensive effects of the EVOO may be due to the phenolic compounds and that they are not a consequence of the fat composition of the oils. However, Kaseb et al., induced a 10.95 mm reduction in supine blood pressure from baseline in hyperlipidemic Iranian volunteers, asked to consume 20 mL of refined olive oil for six weeks. Refined oils are low in polyphenols which would suggest quite significant anti-hypertensive benefits from the lipid fraction of the oil [85].

Human dietary intervention studies with supplemental lipid-free OLE strongly support anti-hypertensive effects for the polyphenols. For example, a study with 40 borderline hypertensive pairs of monozygotic twins discordantly assigned to consume either 500 mg (equivalent to 104 mg OL) or 1000 mg (equivalent to 208 mg OL) of OLE daily for eight weeks was performed by Perrijaquet-Moccetti et al. They observed a dose-dependent drop in SBP, with a mean reduction of 6 mmHg on the 500 mg dose and 13 mmHg on the 1000 mg dose [34]. Elsewhere in a double blind, randomized, parallel and active-controlled clinical study, stage-1 hypertensive patients (*n* = 46) were given either 1000 mg (equivalent to 199 mg OL) aqueous OLE daily or captopril for eight weeks. They observed reductions in SBP and DBP from baseline by −11.5 ± 8.5 and −4.8 ± 5.5 mmHg, respectively for those in the OLE group and by −13.7 ± 7.6 and −6.4 ± 5.2 mmHg, respectively for those in the captopril group [36]. Further, Lockyer et al., performed a randomized placebo-controlled trial with OLE using ambulatory blood pressure as the primary endpoint. In this study 60 volunteers consumed either olive leaf extract, containing 136 mg OL and 6 mg HT, or a control for six weeks prior to crossover. They observed a reduction in 24 h SBP/DBP of about 3 mmHg and following the intervention [38].

These observations are also mirrored in work performed in animal models of hypertension. OLE (15% *w*/*w* OL) when fed to hypertensive rats at 30 mg/kg body weight for five weeks significantly reduced SBP (−21.6 ± 5.5 mmHg) [86]. Khayyal et al. considered the dose response to an OLE (containing 18–26% *w/w* equivalent of OL) in l-NAME (*N*_ω_-Nitro-l-arginine methyl ester hydrochloride) induced hypertensive rats. With daily oral administration of 25, 50 or 100 mg/kg of OLE, they observed a dose-dependent prevention of induced blood pressure with the 100 mg/kg dose completely preventing any rise in blood pressure after eight weeks [87].

The mechanisms of action for olive phenolics in the lowering of blood pressure are uncertain. Lockyer et al. observed a decrease in vascular stiffness relatively soon after the consumption of OLE in an acute human dietary intervention study [33]. This suggests that the phenolics influence production of nitric oxide, thereby improving vascular function in the short term, and with sustained consumption resulting in an improvement in blood pressure over the longer term. Indeed, in vitro, OL has been shown to increase NO production in lipopolysaccharide stimulated mouse macrophages [88], plausibly through the modulation of enzymes such as nicotinamide adenine dinucleotide phosphate-oxidase (NADPH-oxidase) and nitric oxide synthase [89]. Further, OL and HT may act synergistically with other phenolics such as verbascoside to exert angiotensin converting enzyme (ACE) inhibitory [90] and calcium channel blocking activities [91]. However, the phenolics themselves survive digestion relatively poorly; small quantities are present in the circulation as conjugated metabolites [92], and the specific effects of these metabolites on the blood pressure control systems have not been well studied.

### 3.4. Dyslipidaemia

The major components of dyslipidaemia associated with the metabolic syndrome are raised flux of free fatty acids, increased fasting and postprandial triglyceride-rich lipoproteins (TRLs), decreased high-density lipoprotein (HDL), and increased small, dense low-density lipoprotein (LDL) particles [93]. Exaggerated postprandial lipaemia links MetS to the progression of atherosclerosis [76]. Olive oil with its high MUFA content is shown to reduce TC and LDL-C levels when substituted into the diet in place of saturated fats [94]. The additional beneficial effects of the phenolics in EVOO have also been studied [10]. In a large multi-centre crossover trial in healthy men (*n* = 200), Estruch et al. demonstrated the dose dependent improvements in plasma HDL status in response to increasing polyphenol concentrations in olive oils [95]. Several human chronic dietary intervention studies with aqueous olive leaf extracts have also demonstrated favourable plasma lipid responses. Perrinjaquet-Moccetti et al., Sasulit et al., Lockyer et al. and Fonollá J et al. all observed significant reductions in total cholesterol, LDL-cholesterol and triglyceride levels in human volunteers [34,35,36,38]. In contrast De bock et al. did not observe improvements in lipid profiles after OLE supplementation [32]. In each of these studies plasma lipids were measured as secondary endpoints; therefore more focussed human studies are needed to fully elucidate potential benefit of the phenolics independently of the oil in relation to dyslipidaemia.

In rodent models, chronic OLE supplementation led to a reduction in total cholesterol, LDL-C and triglycerides [54,96,97,98]. Further Ghosian Moghaddam et al. observed an improvement in HDL-C in diabetic wistar rats fed powdered olive leaf mixed into chow at 6.25% *w*/*w* [99] and Jemai et al. observed protection against dyslipidaemia in cholesterol fed rats for both HT and an OL-rich olive leaf extract [72,100].

The evidence for the anti-hyperlipidaemic effects of olive phenolics is not as strong as it is for the anti-hypertensive and anti-diabetic effects, nevertheless potential mechanisms action have been postulated. In the small intestine, phenolics may inhibit pancreatic lipases, thus delaying post-prandial lipaemia [101], Further better glucose and insulin control would reduce the accumulation of lipids in the liver, as observed in a cholesterol fed rat model [100], and potentially offset de-novo lipogenic pathways.

### 3.5. Lipid Peroxidation

The oxidation of LDL is a free radical driven process that is believed to stimulate the macrophage uptake of lipoproteins, and Subsequently, to induce foam cell formation and inflammatory responses [102], subsequently oxidized LDL is associated with increased incidence of metabolic syndrome and coronary heart disease [103].

There are consistent data on the effects of extra virgin olive oil on markers of lipid peroxidation. The European Food Safety Authority now accepts health claims concerning the effectiveness of the ingestion of olive oil with high concentrations of HT and its derivatives (5 mg/day) at suppressing lipid peroxidation [13,45,48,95,104]. It has been assumed that in vitro the conferred protection against oxidative stress is mediated by HT and its derivatives [105], however this hypothesis has never been confirmed in humans [106]. Further, in an eight week long chronic intervention trial with 45 mg per day pure hydroxytyrosol, Lopez-Huertas et al., observed no significant reduction in oxidised LDL. They did however report elevated serum vitamin C in cases versus controls, suggesting the sparing of vitamin C for antioxidant function with HT consumption [107].

Only one human dietary intervention study with a lipid free olive leaf extract that demonstrated reduced LDL oxidation is reported in the literature, with a 13% reduction in oxidized LDL reported in hypercholesteraemic subjects, and this was only presented in conference abstract form [35]. Protection against LDL oxidation for lipid free olive extracts is observed in animal models. Wistar rats fed a cholesterol-rich diet in combination with OLE for 16 weeks showed reduced LDL oxidation, in combination with an upregulation of antioxidant enzymes [100]. Elsewhere, mice fed 15% olive oil for six weeks had demonstrably reduced lipid peroxidation compared to the controls [108]. Rabbits fed with 10% (*w*/*w*) extra virgin olive oil plus 7 mg/kg of OL were protected against LDL oxidation [109], and Wistar Rats fed a high energy diet showed a reduction in markers of oxidative stress when given a 20 µg daily oral gavage with HT [66].

In vitro, olive polyphenols inhibit the copper sulphate-induced oxidation of LDL [110] and counteract both metal- and radical-dependent LDL oxidation [111]. The direct anti-oxidant actions of polyphenols delivered through a routine diet is likely to be considerably lower in vivo given their relatively low abundance, bioavailability and rapid clearance. Giordano et al. demonstrate a down regulation of the oxidative stress pathways in mouse adipocytes post HT consumption [67]. The influence of olive polyphenolics on the Nrf2 pathway in the combatting of oxidative stress has recently been reviewed by Piroddi et al., whilst not yet having been proven in humans; this may turn out to be a viable mechanistic explanation for the antioxidant responses to olive polyphenol consumption [112].

## 4. Discussion

In this review, we have summarised the findings of the dietary intervention studies with olive oil or with lipid free olive leaf extracts, which have explored aspects of the MetS.

**Figure 1:**
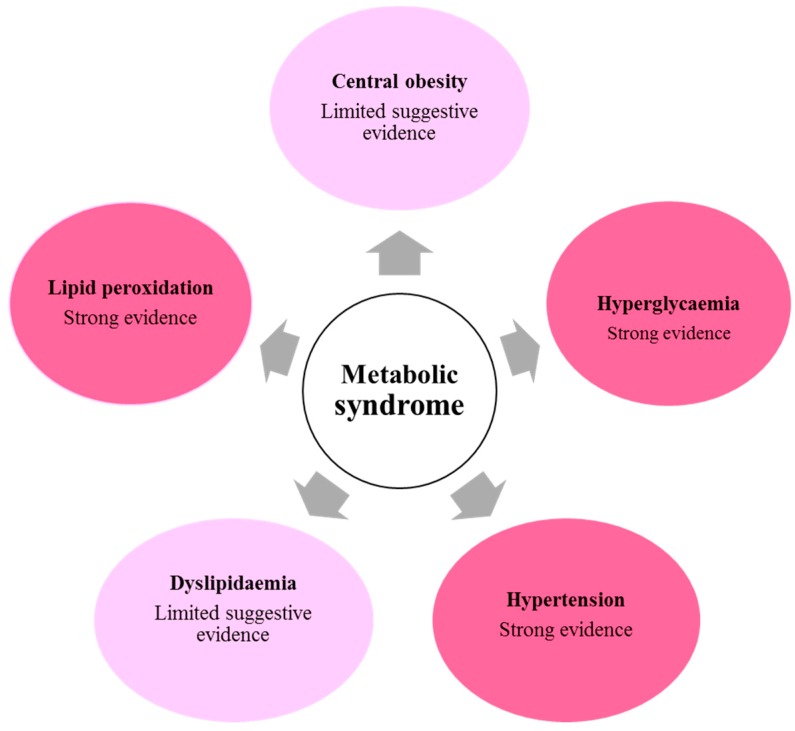
Summary of the quality of evidence from intervention studies demonstrating the influence of olive polyphenols on aspects of the metabolic syndrome.

To summarise the evidence for benefits directly attributable to olive phenolics coming from human dietary intervention studies is strongest for the observed protection against the oxidation of lipoproteins, protection against hypertension, and protection against hyperglycaemia. There is limited, but suggestive evidence for effects on dyslipidaemia and the inhibition of weight gain (Figure 1). Further dietary intervention studies in humans in which the primary outcome measures are dyslipidaemia or adiposity are needed.

In an obese age, the consumption of phenolic-rich, lipid-free, valorised olive leaf products may be a preferential approach to ingesting these beneficial compounds. Intervention studies with EVOO, consumed in quantities of up to 50 g per day, delivered an estimated 5–20× lower dose of total phenolics than were consumed in intervention studies with the olive leaf extract supplement. Directly replacing the traditional 10 g per day of culinary spreads and dressings consumed as part of the UK diet with EVOO would beneficially increase the ratio in the intake of MUFA’s relative to SFA’s, but it would not substantially change the total polyphenol intake. The PREDIMED (Primary Prevention of Cardiovascular Disease with a Mediterranean Diet) intervention trial clearly demonstrates the efficacy of a Mediterranean pattern of food consumption over a low fat diet, but does not show a reduction in mortality with an EVOO enriched Mediterranean compared to that observed for the same diet supplemented with nuts instead of EVOO [113].

A current weakness of the available evidence is the paucity of data on mechanisms of action; approximately 60% of ingested olive oil phenols are absorbed in humans, mainly in the small intestine, and peak, as derivatives, in ng mL^−1^ concentrations, in plasma 1–2 h post consumption [114]. During digestion, and with metabolism, oleuropein is hydrolysed, and later recovered in urine, primarily as glucuronides of hydroxytyrosol and tyrosol [92,115]. In vitro, mechanistic studies have typically evaluated the effects of the un-metabolized major phenolics at supra-physiological doses in cell systems which are often poorly validated against the in vivo system, The effects of the minor phenolics are also comparatively under-assessed. Genomic and transcriptomic studies in animal models point to the activation of pathways involved in inflammation, oxidative stress and macronutrient metabolism as potential mechanisms of action [116], However further molecular nutrition based research should complete our understanding of the interactions between the bioactive components of olive polyphenols.

## 5. Conclusions

There is good evidence from human intervention studies showing that, when consumed at appropriate doses, olive polyphenols induce a marked reduction in blood pressure in hypertensive subjects. They also induce an improvement in blood glucose in prediabetes, and improvements in markers of lipid peroxidation. The required doses are above those consumed habitually in most diets. We would, however, caution against advocating the use of very large quantities of olive oil given its potential contribution to energy intake. Evidence is needed to confirm the interactions and combined benefits of olive polyphenols and other plant polyphenols in the context of the Mediterranean pattern of food intake, but they may, in part, explain some of the myriad of metabolic benefits associated with that diet.

## Figures and Tables

**Table 1:** Summary of key human studies showing the effectiveness of olive leaf extracts (OLE) and extra virgin olive oils (EVOO) on the metabolic syndrome (MetS) and associated risk factors.

Source	Intervention	Participants	Dose	Main Finding				References
				Central Obesity	Hyperglycaemia	Hypertension	Hyperlipidaemia	
Olive leaf extract	A randomised, double-blind, placebo-controlled, cross-over, acute intervention trial	18 male and female (healthy volunteers)	1600 mg OLE (51.12 mg OL and 9.67 mg HT)	na	na	↓DVP-SI	↓IL-8	[33]
Olive leaf extract	Eight weeks, An open, controlled, parallel-group, co-twin study	40 monozygotic twins (pre-hypertensive subjects)	500 mg OLE/day (104 mg OL/day) or 1000 mg OLE/day (208 mg OL/day) (control group receiving no medication)	ns	ns	↓SBP ↓DBP	↓LDL	[34]
Olive leaf extract	28 days, A longitudinal, controlled, randomized, and double-blind intervention study	39 male and female (hyperlipidaemia subjects)	1200 mg OLE/day (control group receiving placebo)	na	na	na	↓TC ↓LDL ↓ox-LDL ↓TC/HDL ratio ↓TAG	[35]
Olive leaf extract	Eight weeks, A double-blind, randomized, parallel and active-controlled clinical study	148 male and female (stage-1 hypertensive subjects)	1000 mg OLE/day (control group receiving Captopril 12.5 mg)	na	na	↓SBP ↓DBP	↓TC ↓LDL ↓TAG	[36]
Olive leaf extract	14 weeks, A randomized, double-blind, placebo controlled, clinical trial	79 male and female (Type II diabetic subjects)	500 mg OLE/day (control group receiving placebo)	ns	↓HbA1C	ns	ns	[37]
Olive leaf extract	30 weeks, A randomized, double-blinded, placebo controlled, crossover trial	46 male (overweight subjects)	OLE/day (51.1 mg OL and 9.7 mg HT)	ns	↑insulin sensitivity	ns	ns	[32]
Olive leaf extract	16 weeks, A randomized, double-blinded, placebo controlled, crossover trial	61 male (overweight subjects)	20 mL OLE/day (136.2 mg OL/day)	ns	ns	↓24 h SBP ↓24 h DBP ↓Daytime SBP ↓Daytime DBP	↓TC ↓LDL ↓TAG	[38]
Olive oil (unrefined)	Three months, A randomized, two-period, crossover design	24 male (peripheral vascular disease subjects)	Replacement of culinary oils with extra virgin olive oil (control group replacing with refined olive)	ns	na	na	↓ox-LDL	[39]
Olive oil (unrefined)	Six months, A double-blind, randomized, crossover study	23 male and female (hypertensive patients)	30–40 g of oil per day (control group receiving sunflower oil)	ns	ns	↓SBP ↓DBP	ns	[40]
Olive oil (unrefined)	30 days, A randomized crossover trial	25 male and female (healthy subjects)	10 g of extra virgin olive oil (control group receiving corn oil)	na	↓blood glucose ↑insulin	na	↓ox-LDL	[13]
Olive oil (unrefined)	Three months, A randomized, single-blinded and placebo-controlled trial	41 male and female (overweight or obese subjects)	Replacement of culinary oils with extra virgin olive oil (control group replacing with 10% corn oil and 90% soybean oil)	ns	ns	↓SBP	↑HDL	[41]
Olive oil (unrefined)	One year A randomized, placebo-controlled trial	351 male and female (type 2 diabetes or ≥ three cardiovascular disease (CVD) risk factors)	MeDiet + extra-virgin olive oil (control group receiving MeDiet + nuts (walnuts, almonds, and hazelnuts), or a control low-fat diet.	↓body weight ↓BMI ↓WC ↓body fat distribution	na	na	na	[42]
Olive oil (unrefined)	Five years, A parallel-group, multicenter, randomized clinical trial	7447 male and female (type 2 diabetes or ≥Three CVD risk factors)	MeDiet + 50 mL extra-virgin olive oil (control group receiving MeDiet + 30 g nuts (walnuts, almonds, and hazelnuts), or a control low-fat diet.	↓body weight ↓central adiposity	na	na	na	[43]
Olive oil (unrefined)	Three weeks, A double-blind, crossover, randomized, controlled clinical trial	30 male and female (healthy subjects)	25 mL of virgin olive oil over three meals per day (control group receiving 25 mL of refined oilve oil per day)	ns	ns	na	↑HDL ↓ox-LDL	[44]
Olive oil (unrefined)	Three weeks, A randomized, double-blinded, placebo controlled	40 male (hypertensive subjects)	50 mL of virgin olive oil per day (control group receiving 50 mL of refined oilve oil per day)	ns	ns	↓SBP	↓ox-LDL ↓Lipoperoxides	[45]
Olive oil (unrefined)	Five weeks A randomized, two-period, crossover design	200 male (healthy volunteers)	25 mL of virgin olive oil per day (control group receiving 25 mL of refined oilve oil per day)	ns	ns	ns	↑HDL ↓TAG ↓ox-LDL	[46]
Olive oil (unrefined)	Three years, A randomized dietary trial	187 male and female (metabolic syndrome subjects)	MeDiet + extra-virgin olive oil (control group receiving MeDiet + nuts (walnuts, almonds, and hazelnuts), or a control low-fat diet.	↓body weight ↓WC	na	na	na	[47]
Olive oil (unrefined)	Four months, A double-blind, randomized, crossover dietary intervention trial	24 female (mind hypertensive subjects)	30 mg/day of polyphenols from olive oil (control group receiving polyphenol-free olive oil)	na	ns	↓SBP ↓DBP	↓ox-LDL	[48]

↓↑, significant augmentation or diminution; ns, not significant; na, not assayed or not reported; BMI, body mass index; WC, waist circumference; DVP-SI, digital volume pulse-stiffness index; SBP, systolic blood pressure; DBP, diastolic blood pressure; LDL, low density lipoprotein; HDL, high density lipoprotein, ox-LDL, oxidized low density lipoprotein; TAG, triacylglycerol.

## Supplementary Materials

Supplementary File 1
