# Supplementary material for: Olive Polyphenols and the Metabolic Syndrome
Source: Molecules. 2017 Jun 29;22(7):1082. doi: 10.3390/molecules22071082 (PMC6152042; doi:10.3390/molecules22071082)
Supplement: Supplementary File 1 [file molecules-22-01082-s001.pdf]

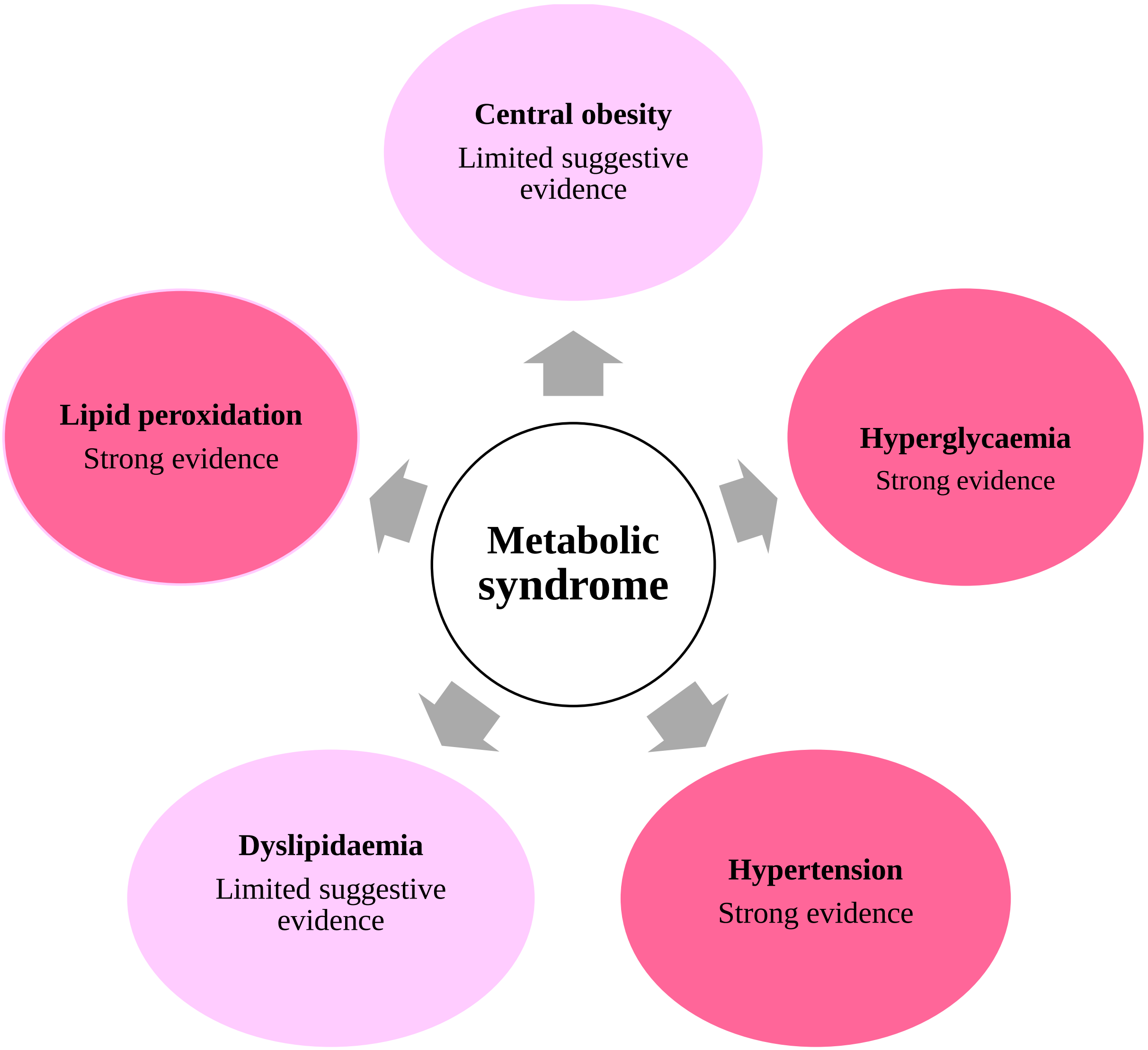

Figure 1 Influence of olive polyphenols on reducing clinical risk factors in the metabolic syndrome.
